# Good neighbors? Does aggregation of nests in an Arctic‐breeding shorebird influence daily survival rates?

**DOI:** 10.1002/ece3.10137

**Published:** 2023-06-21

**Authors:** Scott L. Freeman, Katelyn M. Luff, Kirsty E. B. Gurney

**Affiliations:** ^1^ Montana Fish, Wildlife & Parks Kalispell Montana USA; ^2^ Water Security Agency, Ecological and Habitat Assessment Saskatoon Saskatchewan Canada; ^3^ Department of Biology University of Saskatchewan Saskatoon Saskatchewan Canada; ^4^ Science & Technology Branch, Environment and Climate Change Canada (ECCC) Saskatoon Saskatchewan Canada

**Keywords:** *calidris*, habitat, nest density, Nunavut, semipalmated sandpiper

## Abstract

Our current understanding of the factors that influence where birds nest is incomplete, yet such information is important for accurate demographic assessments. To address questions related to spatial distributions of shorebird nests and to evaluate factors that may affect nest distribution in these species, during 2017 and 2019, we studied a small population of semipalmated sandpiper *Calidris pusilla* breeding in the Central Canadian Arctic, near the Karrak Lake Research Station, in Nunavut. The spatial distribution of semipalmated sandpiper nests at this site suggested loose aggregation, with median nearest neighbor distances of 73.8 m and 92.0 m in 2017 and 2019, respectively, while no nests were detected on mainland areas in the vicinity. Evidence for the influence of nesting distribution on the daily survival rate of nests, however, was mixed. Neither nearest neighbor distance nor local nest density had a significant effect on daily nest survival in 2017, but in 2019, the best approximating model included an effect of local nest density, which indicated that nests in areas of high density had reduced survival rates. Contrary to other studies assessing settlement and nest site selection in semipalmated sandpipers, the spatial distribution of nests in this population demonstrates aggregation in an otherwise territorial species, but suggests that aggregated nesting can impose a cost on nest survival under certain conditions.

## Graphical abstract

Semipalmated sandpipers are monogamous and territorial and are expected to avoid nesting in close proximity to conspecifics. We study an aggregated population nesting in the Canadian Arctic and test for the impacts of nest density and nearest neighbor distance on nest success.

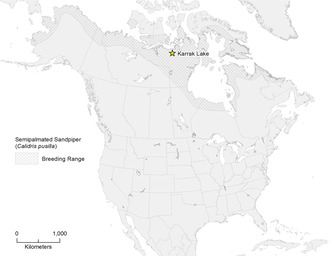

## 1 INTRODUCTION

Across avian taxa, the spatial distribution of nests ranges from dispersed and solitary to extremely dense aggregations. Colonial nesting (i.e., aggregated nesting regardless of cooperative behavior; Siegel‐Causey & Kharitonov, 1990) has evolved independently several times, yet remains relatively uncommon in birds, occurring in only about 13% of species (Lack, 1968; Rolland et al., 1998), where benefits of breeding in close proximity to conspecifics outweigh potential costs, such as food depletion or increased conspecific brood parasitism (Bonal & Aparicio, 2008; Craik et al., 2018). Benefits of aggregations may include reduced predation due to group defence, increased opportunities for extra‐pair copulations, or increased food‐finding efficiency (Evans et al., 2016; Lengyel, 2006; Mayer & Pasinelli, 2013). Aggregated nesting can also be the result of habitat selection, wherein individuals aggregate in response to a patchy distribution of resources (Pérot & Villard, 2009). Although identifying selective mechanisms for the evolution of coloniality has been the focus of intensive study, ultimate factors leading to, and maintaining, aggregated nesting appear to be highly context dependent, such that benefits associated with nesting in groups often remain unclear (Di Maggio et al., 2013; Tarof et al., 2004; Varela et al., 2007).

Understanding ultimate factors that affect nesting distributions, however, is central to the successful management of wild birds, particularly given the potential additive effects of proximate factors—and thus ongoing environmental change—on distributions of free‐ranging animals (Bonter et al., 2014; Wauchope et al., 2017; Winnicki et al., 2020). Further, conspecific attraction in the selection of breeding sites has important implications for estimates of population size, and the effectiveness of density as an indicator of habitat quality is reduced when birds are attracted to breeding areas in part due to the presence of conspecifics (Jehl, 2006; Skagen & Yackel Adams, 2011). Population estimates, and the assessment of other conservation metrics like habitat quality, may therefore be biased if they do not account for clustering of nests within habitat, and such biases will be particularly important for species in decline and those of conservation concern (Byerly et al., 2021; Pérot & Villard, 2009). Many Arctic‐nesting shorebird species, for example, are experiencing population declines (Hope et al., 2019; Rosenberg et al., 2019), but whether aggregated nesting occurs in these species—and potential costs and benefits have received limited attention (Johnson & Walters, 2011).

Patterns of spatial distributions for shorebird nests vary across species, with dispersed, solitary nesting being the most common; fewer species nesting in either small groups or low‐density aggregations; and a limited number of species that nest at high densities (Larsen et al., 1996), potentially due to limited benefits of coloniality for ground‐nesting species (Page et al., 1983 but see Macdonald & Bolton, 2008). Aggregated nesting among shorebirds is most commonly found in larger species, which benefit from group mobbing of predators (Berg, 1996; Kirby, 1984; Takahashi & Ohkawara, 2007). Although clustering is observed in some smaller shorebird species, the benefits are unclear (Armstrong & Nol, 1993; Fraga & Amat, 1996; Nelson, 2007; Rae et al., 1998). Aggregated nesting in smaller species could provide opportunity for extra‐pair copulations, but extra‐pair paternity tends to be relatively low in shorebirds and is found primarily in species with low rates of philopatry and mate fidelity (Küpper et al., 2004; Wagner, 1998; Yezerinac et al., 2013).

Aggregated nesting is therefore not expected among monogamous and typically territorial calidrine sandpipers (Pitelka et al., 1974). Rather, for such species, nests should be distributed relatively uniformly within available habitats (Saalfeld & Lanctot, 2015), particularly because dispersed breeding can be advantageous for smaller species which cannot defend themselves against predators (Colwell, 2010; Ellis et al., 2020). Empirical evidence supporting this idea, however, is equivocal. Nesting densities among semipalmated sandpipers can be high, and in some cases, described as colonial (Jehl, 2006), but internest distances for semipalmated sandpipers in Alaska were larger than expected assuming random placement of nests, which suggests avoidance of conspecifics (Cunningham et al., 2016). As such, the extent of aggregated nesting in this small calidrine species is unclear and potential ultimate factors contributing to aggregation remain unexplained.

To address questions related to spatial distributions of shorebird nests and to evaluate factors that may influence nest distributions in these species, we collected field data over 2 years in the Central Canadian Arctic, at the Karrak Lake Research Station, in Nunavut. Our specific objectives were (i) to describe the spatial distribution of semipalmated sandpiper nests at this site and (ii) to test for impacts of spatial distribution on the daily survival rate of nests. Semipalmated sandpiper (hereafter sandpiper) at Karrak Lake are ideal models for studying nesting dispersion, as the relatively high density of their nests within limited habitat at this site suggests conspecific attraction in an otherwise territorial species. Further, the Central Canadian Arctic is an understudied part of the sandpiper breeding range and, from a conservation standpoint, demographic studies in this area warrant further attention (Andres et al., 2012; Smith et al., 2012).

Previous studies report contrasting relationships between spatial distribution of shorebird nests and nest survival. Among small‐bodied shorebirds in particular, nest density may be either uncorrelated with nest success (Patrick, 2013), correlated with higher nest success (Armstrong & Nol, 1993) or conversely, may be correlated with higher rates of depredation and reductions in nest survival (Catlin et al., 2019; Ellis et al., 2020; Page et al., 1983), possibly due to differences in predator communities across space and time (Brown et al., 2022). At Karrak Lake, where there are active dens of a key shorebird predator, the Arctic fox *Vulpes lagopus* (Mckinnon & Bêty, 2009), we predicted that aggregated nesting by sandpipers would impose a cost (our cost of aggregation hypothesis), resulting in a positive relationship between nest survival and nearest neighbor distance.

## 2 METHODS

### 2.1 Study area

**FIGURE 1:**
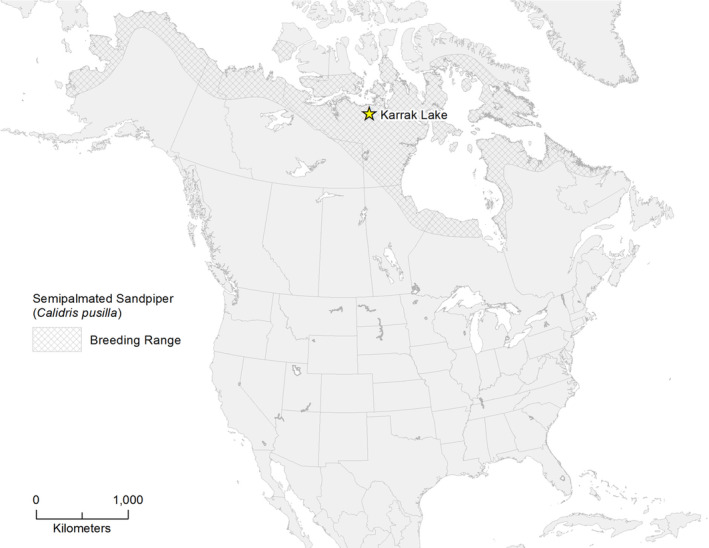
Karrak Lake Research Station, in the Central Canadian Arctic, is located in the Ahiak Migratory Bird Sanctuary, south of the Queen Maud Gulf.

The sandpiper nests are located in the Ahiak Migratory Bird Sanctuary, near Karrak Lake Research Station, on the largest of several islands (Camp Island) in Karrak Lake, Nunavut (Figure 1). Camp Island has an area of approximately 1.5 km^2^ and is covered primarily with rock and gravel, low‐growing vegetation such as dwarf birch or graminoids, and small ponds and wetlands. High‐density nesting of light geese—lesser snow goose *Anser caerulescens caerulescens* and Ross's goose *Anser rossii*—at the site dates back to the 1960s or earlier and has led to the modification of the vegetation of Camp Island through the removal of graminoids from low lying areas and subsequent encroachment by dwarf birch and Labrador tea (Alisauskas et al., 2006). Light geese still nest on Camp Island, but nest density of these species is now lower than historical levels (Kellett, 2021), as the area of concentrated nesting has shifted to the north. In addition to light geese and sandpipers, other avian species nesting on the island include Canada and cackling geese *Branta canadensis, B. hutchinsii hutchinsii*, king eider *Somateria spectabilis*, Arctic tern *Sterna paradisaea*, sandhill crane *Antigone canadensis*, horned lark *Eremophila alpestris*, Lapland longspur *Calcarius lapponicus*, and semipalmated plover *Charadrius semipalmatus*. The lone Arctic fox den on Camp Island was active in 2019 but not in 2017.

### 2.2 Data collection

**FIGURE 2:**
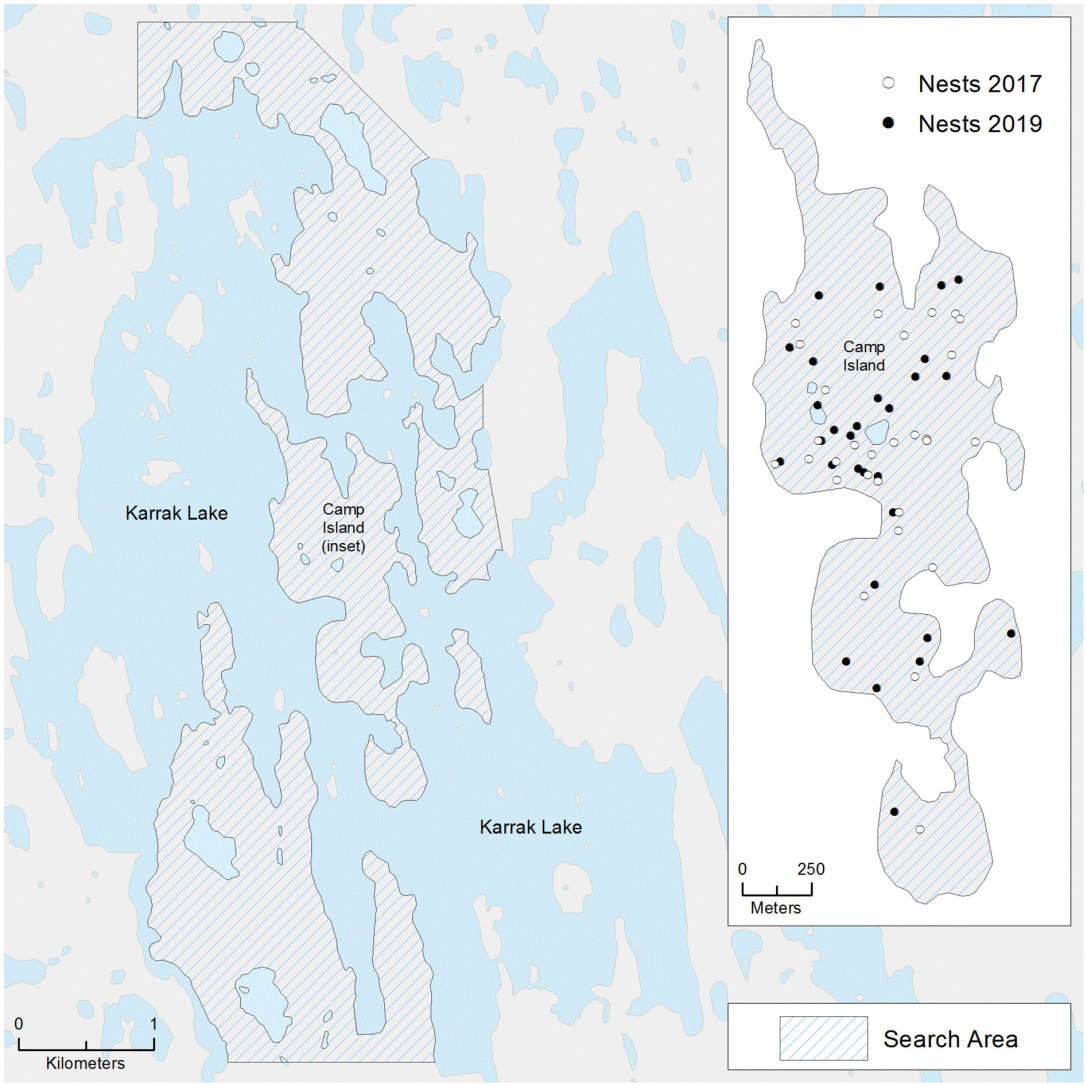
Location of semipalmated sandpiper nests in the vicinity of Camp Island, Karrak Lake, Nunavut. Nests are coded by year (2017 = black, 2019 = gray). Search areas are shown by cross‐hatching.

Throughout the nesting season (early June–early July), we searched intensively (three dedicated people in 2017 and one person in 2019) for sandpiper nests on Camp Island and on mainland areas in the vicinity of Karrak Lake (Figure 2). We detected nests either by flushing birds from a nest or by observing birds returning to a nest. We made notes on locations of male territorial displays and interactions between apparently mated pairs and returned to these areas repeatedly to search for nests. Nest locations were recorded using a handheld Global Positioning System device and marked with a popsicle stick placed 5 m from the nest, as well as a pin flag placed an additional 5 m from the nest. For partial clutches, we determined nest age based on observed laying dates, and for full clutches, we used flotation methods and back‐calculated the nest initiation date from the estimated incubation date (Liebezeit et al., 2007). If nests were found late in incubation, and if the nest hatched, we back‐calculated the nest initiation date from the hatch date, using an estimated incubation period of 20 days (Hicklin & Gratto‐Trevor, 2020).

We revisited nests every 5 days until nests were within 4 days of the estimated hatch date. Four days before the predicted hatch, we checked all eggs for evidence of hatch (cracks, stars, or pips evident on at least one egg). If evidence of hatch was observed, we revisited the nest every day until nest completion. If no evidence of hatch was observed 4 days before the predicted hatch, then we revisited the nest 2 days before the predicted hatch and every day thereafter. For nests found prior to the hatching period, we used a remotely triggered bow net to capture birds on the nest (Priklonsky, 1960). Captured birds were banded (one aluminum band and one plastic colored band each) and measured (culmen length, total head, tarsus length, and mass). As part of another study, we placed temperature loggers in a subset of nests (*n* = 14) to evaluate incubation constancy (Lunny, 2019), with visits for trapping, temperature logger deployment, and logger retrieval augmenting the regular nest visit schedule. Nest fate was assigned using criteria developed by the Arctic Shorebird Demographics Network (Brown et al., 2014). When we suspected nest abandonment, we rearranged one egg with the pointed side out and recorded the egg position on that and the subsequent visit. Nests with inconclusive evidence were classified as “of unknown fate.”

Hourly average temperature data were collected by an automated weather station (Campbell Scientific CR800) maintained on Camp Island. To index the melting of snow, in 2019, all nest sites from 2017 were visited by an observer on the date of first nest initiation in that year (7 June). Each nest site was categorized as either covered in snow or snow free.

### 2.3 Statistical analyses

All analyses were implemented in program *R* ver. 3.6.1 (Action of the Toes, R Core Team, 2019). For a general comparison of overall nest density to other studies we divided the number of detected nests by the entire surface area of Camp Island, by year, without correcting for areas of surface water on the island or for other areas that might not be suitable nesting habitats. Nearest neighbor distances (including all nests regardless of initiation date) were determined using the “pointDistance” function in the package “raster” in program *R* (Hijmans et al., 2020), and local nest density was calculated as the number of conspecifics within the median distance between nests. Daily nest survival analyses—based on linear exposure methods—were conducted using the “glm” function in the “stats” package in program R (Shaffer, 2004). The link function used in these analyses was adapted from Shaffer by Mark Herzog and was further developed in code published as an RMarkdown document on RPubs by Ben Bolker (https://rpubs.com/bbolker/logregexp).

We began by developing an a priori set of candidate models that represented daily survival rate (DSR) as a function of additive combinations of predictor variables that were selected based on our “cost of aggregation” hypothesis and the results of previous investigations. The main predictor variables of interest included local nest density and nearest neighbor distance. Both statistical (constant DSR) and biological null (intercept and known covariates of nest survival such as nest age, nest age^2^, day of year, and day of year^2^) models were included in our candidate set, and to test for cumulative impacts of additional human activities near nests, the presence of a temperature logger was included as a potential predictor. Models with additive combinations of explanatory variables that were correlated (nearest neighbor and local density; nest age and season day) were not considered, and all numerical predictor variables were standardized (converted to *z*‐scores) to deal with potential issues related to differing scales (Schielzeth, 2010). For model testing, we evaluated the 2 years of data separately, censoring one nest with erroneous location data in 2017 and two nests that were not aged before failure (2017, *n* = 30; 2019, *n* = 29).

We used an information‐theoretic approach to model selection, with models ranked according to second‐order Akaike's Information Criterion (AIC_c_) (Burnham & Anderson, 2002) and based inference of effects on the degree to which included variables reduced model deviance (i.e., –2 log likelihood) as well as on the precision (85% CI) of regression coefficients (*β*). If confidence intervals of estimated parameters included zero or were sufficiently wide to indicate a lack of precision, effects associated with these parameters were considered to be uninformative. Models containing such effects were not used for inference (Arnold, 2010).

Finally, we estimated annual nest success from combined annual data and included some nests that lacked covariate measures used in model testing (*n* = 62 nests). The probability that a nest survived the nesting period (nest success) for each year was estimated as DSR^n^, where n is the normal nesting period for sandpipers (lay period of 3 days plus incubation period of 20 days). Confidence intervals for DSR were back‐transformed from the 85% confidence interval (CI) on the logit scale, and nest success CIs were calculated using the Delta method (Powell, 2007).

## 3 RESULTS

The small size of the study area facilitated intensive nest searching, such that across both years, 26 of 62 sandpiper nests (42%) were discovered either with a partial clutch or on the estimated day of clutch completion. Although mainland areas are separated from Camp Island only by narrow stretches of water (within <200 m to the north, east, and southwest) and have similar habitats to that found on Camp Island, we detected no nests on mainland areas in the vicinity of Camp Island.

In 2017, nest initiation dates ranged from June 5 to June 25 (median June 10), including one re‐nest by a pair that used the same nest cup after the failure of their first nest. In 2019, nest initiation dates were later, ranging from June 11 to July 4 (median June 20). The mean average daily temperature between the last week of May and the first 2 weeks of June was 2.8°C in 2017 (standard deviation, SD = 1.62) and − 1.1°C in 2019 (SD = 2.95), with 48% of the 2017 nesting sites covered or partially covered with snow on June 7, 2019.

### 3.1 Spatial distribution of nests

No nests were detected on mainland areas in the vicinity of Camp Island, and nesting by sandpiper on Camp Island was concentrated in the central portion of the island and confined to shrub or graminoid habitat that provided nest cover (Figure 2). Annual nest density estimates for Camp Island were 21.3 and 20.0 nests/km^2^ for 2017 and 2019, respectively. In 2017, nearest neighbor distances ranged from 8 to 555 m, with a median value of 73.8 m (SD = 110.4). In 2019, nearest neighbor distances ranged from 24 to 455 m, with a median of 92.0 m (SD = 95.1). In 2017, the maximum local nest density was 135.0 nests/km^2^ with a median of 27.0 nests/km^2^ (SD = 35.5). In 2019, the maximum local nest density was 87.2 nests/km^2^ with a median of 21.8 nests/km^2^ (SD = 26.2). Combining both years, 12 of 61 nests (20%) were within 50 m of a conspecific nest.

### 3.2 Nest survival

**FIGURE 3:**
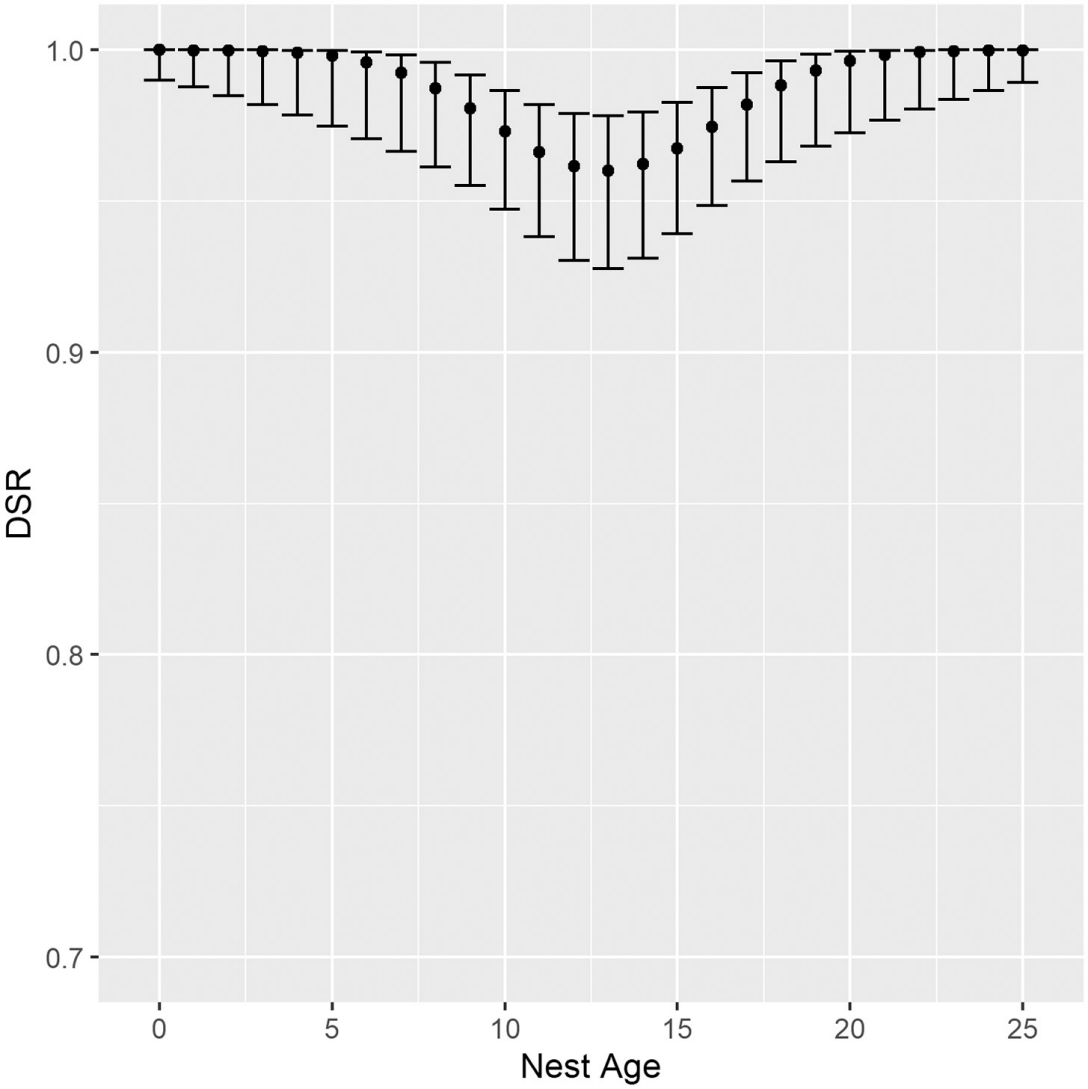
Daily survival rate (DSR) of nests from the model S ~ Nest Age + Nest Age^2^ for shorebirds nesting at Karrak Lake, Nunavut, in 2019. Confidence intervals (85%) are back‐transformed from the standard error estimates on the logit scale.

**FIGURE 4:**
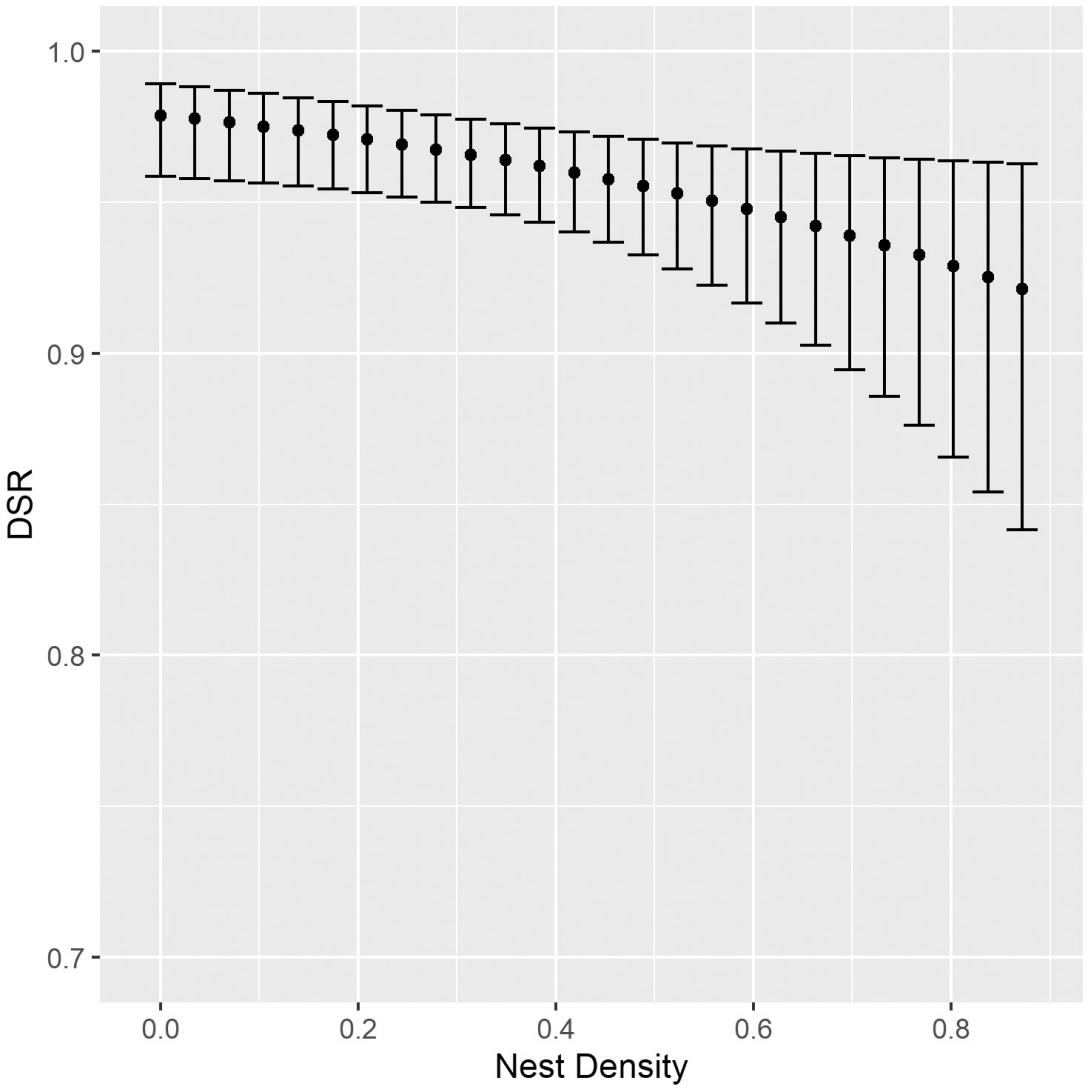
Daily survival rate (DSR) of nests from the model S ~ Density for shorebirds nesting at Karrak Lake, Nunavut, in 2019. Confidence intervals (85%) are back‐transformed from the standard error estimates on the logit scale.

For 2017, contrary to our expectations, our best‐supported model did not include an effect of nearest neighbor distance or local nest density on predicted DSR (Table 1), but rather indicated lower nest success for nests in mid‐incubation (*β*_NestAge_ = −.178; 85% CI: −1.158–0.803; *β*_NestAge_^2^ = 1.627; 85% CI: 0.299–2.956) (Akaike weight, *w*_*i*_ = 0.17, Table 2, Figure 3). Conversely, for 2019, our best‐supported model did not include either linear or quadratic temporal terms (Table 3) but did include a density term, which indicated reduced nest success with increasing local nest density (*β*_Density_ = −.442; 85% CI: −0.834 to −0.049) (Akaike weight, *w*_*i*_ = 0.18, Table 4, Figure 4). There is low support for models with logger terms. Estimates of annual nest survival probabilities were 0.647 (85% CI: 0.513–0.781) in 2017 and 0.412 (85% CI: 0.269–0.556) in 2019.

**TABLE 1:** Ranking of daily survival models for semipalmated sandpiper nests at Karrak Lake, Nunavut in 2017 (*n* = 30 nests). Models are ranked by differences in Akaike's Information Criterion, corrected for sample size (ΔAIC_c_).

Model	K^a^	Deviance	ΔAIC_c_	*w*_*i*_	Cumulative *w*_*i*_
NestAge + NestAge^2^	3	64.086	0.000	0.172	0.172
Day+Day^2^	3	64.550	0.464	0.137	0.309
Day+Day^2^ + Logger	4	64.736	0.650	0.125	0.434
NestAge+NestAge^2^ + Density	4	65.893	1.807	0.070	0.503
NestAge+NestAge^2^ + Logger	4	65.937	1.851	0.068	0.572
Day+Day^2^ + NearN	4	66.038	1.952	0.065	0.637
NestAge+NestAge^2^ + NearN	4	66.114	2.028	0.063	0.699
Constant DSR	1	66.292	2.206	0.057	0.756
Day + Day^2^ + Density	4	66.530	2.443	0.051	0.807
Day	2	67.318	3.232	0.034	0.841
Logger	2	68.014	3.928	0.024	0.866
Density	2	68.203	4.117	0.022	0.888
NestAge	2	68.289	4.203	0.021	0.909
NearN	2	68.293	4.207	0.021	0.930
Day + Logger	3	68.438	4.352	0.020	0.949
Day + NearN	3	69.163	5.076	0.014	0.963
Day + Density	3	69.341	5.255	0.012	0.975
NestAge + Logger	3	70.038	5.951	0.009	0.984
NestAge + Density	3	70.197	6.111	0.008	0.992
NestAge + NearN	3	70.304	6.218	0.008	1.000

Abbreviations: NearN, nearest neighbor distance; *w*_*i*_, Akaike weight.

^a^ Number of parameters estimated.

**TABLE 2:** Parameter estimates and standard errors for Nest + Nest age^2^ model for effects on daily survival of semipalmated sandpiper nests at Karrak Lake, Nunavut, in 2017.

Coefficients	Estimate	SE	85% CI	z value	pr (>|*z*|)
Intercept	3.183	0.435	2.556, 3.810	7.329	2.32E‐13
Nest age	−0.178	0.681	−1.158, 0.803	−1.484	0.1377
Nest age^2^	1.627	0.923	0.299, 2.956	1.764	0.0777

**TABLE 3:** Ranking of daily survival models for semipalmated sandpiper nests at Karrak Lake, Nunavut in 2019 (*n* = 29 nests). Models are ranked by differences in Akaike's Information Criterion, corrected for sample size (ΔAIC_c_).

Model	K^a^	Deviance	ΔAIC_c_	*w*_*i*_	Cumulative *w*_*i*_
Density	2	86.653	0.000	0.178	0.178
Constant DSR	1	87.138	0.485	0.140	0.318
NestAge+Density	3	88.063	1.409	0.088	0.406
NearN	2	88.182	1.528	0.083	0.489
Day+Density	3	88.529	1.876	0.070	0.559
Logger	2	88.713	2.060	0.064	0.623
NestAge	2	89.031	2.378	0.054	0.677
Day	2	89.139	2.486	0.051	0.729
NestAge + NearN	3	89.782	3.129	0.037	0.766
NestAge + NestAge^2^ + Density	4	89.826	3.172	0.037	0.803
Day + NearN	3	90.136	3.482	0.031	0.834
Day+Day^2^ + Density	4	90.379	3.726	0.028	0.861
NestAge + Logger	3	90.475	3.821	0.026	0.888
Day + Logger	3	90.729	4.075	0.023	0.911
NestAge + NestAge^2^	3	90.894	4.240	0.021	0.932
Day + Day^2^	3	91.061	4.407	0.020	0.952
NestAge + NestAge^2^ + NearN	4	91.572	4.919	0.015	0.967
Day + Day^2^ + NearN	4	91.955	5.301	0.013	0.980
NestAge + NestAge^2^ + Logger	4	92.296	5.643	0.011	0.991
Day+Day^2^ + Logger	4	92.547	5.893	0.009	1.000

Abbreviations: NearN, nearest neighbor distance; *w*_*i*_, Akaike weight.

^a^ Number of parameters estimated.

**TABLE 4:** Parameter estimates and standard errors for Density model for effects on daily survival of semipalmated sandpiper nests at Karrak Lake, Nunavut, in 2019.

Coefficients	Estimate	SE	85% CI	*z* value	pr (>|*z*|)
Intercept	3.414	0.316	2.959, 3.869	7.329	2.32E‐13
Density	−0.442	0.273	−0.834, −0.049	−1.484	0.1377

## 4 DISCUSSION

Hypothesized causes of aggregated nesting in birds have been extensively studied, particularly with respect to proximate factors, yet how ultimate factors influence nesting distributions remains poorly understood. Here, we examined variation in daily nest survival in an Arctic‐nesting shorebird and tested whether an index of fitness (daily survival probability) was affected either by proximity of the closest conspecific or by density of conspecific nests nearby, thus evaluating a linkage between spatial nesting distribution and an ultimate factor. Although we anticipated that aggregation with conspecifics would increase predation risk and thereby negatively impact the survival probability of nests, our data provide only weak evidence in support of this idea. We did not detect a significant relationship between daily survival rate and distance to the nearest neighbor, and we identified lower daily survival rates in areas of greater nest density only in 2019. Together, these findings suggest that nest survival in this species is affected by aggregation only under certain conditions and that proximate factors, such as habitat quality, or possibly interactions between habitat quality and density, merit further investigation as drivers of nest success in this species.

### 4.1 Spatial distribution of nests

In contrast with mainland areas around Karrak Lake, where no sandpiper nests were found (despite repeated searches), the distribution of nests on Camp Island initially suggested a role for social attraction in an otherwise territorial species (Ashkenazie & Safriel, 1979; Gratto et al., 1985), but our results indicate that other factors, such as habitat selection, may be more important in determining where sandpipers nest. Nests on the island were found predominantly in shrub habitat (dwarf birch and Labrador tea), and although unoccupied habitat of this type is common both on Camp Island and on the surrounding mainland, much of it is either currently, or had recently been, occupied by nesting light geese. Along with other ground‐nesting birds, sandpiper nests are less abundant in areas of high goose density, and nesting aggregations of sandpipers on Camp Island may reflect a combination of selection for birch habitat created by geese before the current study, and avoidance of geese, which can increase the risk of nest predation (Flemming et al., 2019; Lamarre et al., 2017). However, clarifying the extent to which the presence of geese—either through habitat alteration or predation effects—influences nesting distributions of sandpipers at this site will require further study.

Our annual estimates of overall nest density for Camp Island are generally consistent with published estimates of sandpiper nest density (3–300 nests/km^2^), which are highly variable, likely reflecting local and regional differences in nest density, as well as differences in the field and statistical methodologies (Cotter & Andres, 2000; Gratto & Cooke, 1987; Jehl, 2006; Rodrigues, 2002; Saalfeld & Lanctot, 2015). Median nearest neighbor distance, a less commonly reported metric, may be more appropriate for comparing the spatial distribution of nests between study sites, due to greater consistency of methods. Nearest neighbor distances in our study (73.8 and 92.0 m in 2017 and 2019, respectively) illustrate a greater degree of aggregation at Karrak Lake than at Utqiagvik, Alaska (formerly Barrow), where higher values indicate more dispersed nests: 144 m between 2003 and 2012 (Saalfeld & Lanctot, 2015) and 156 m between 2005 and 2012 (Cunningham et al., 2016). Near Churchill, Manitoba, Jehl (2006) reported median nearest neighbor distance that varied from 54.4 to 87.4 m between 1993 and 2004, similar to the values that we found.

In addition to nearest neighbor distances for sandpiper in our study being low compared with values for Utqiagvik, they are also more variable. Whereas sandpipers nesting at Utqiagvik systematically choose nest sites farther from conspecifics than predicted by chance and the standard deviation of nearest neighbor distance is low (Cunningham et al., 2016), our findings indicate that nest site selection at Karrak Lake is not strongly driven by the presence of conspecifics—some individuals nesting in close proximity to neighbors despite the apparent availability of unoccupied habitat at greater distances, and others nesting at much larger distances from neighbors. What might cause variation in nearest neighbor distances over space and time is not clear, but in the Alaskan population, territoriality appears to be a primary driver of nest site selection, whereas at Karrak Lake other factors, such as habitat selection or philopatry, may result in greater variation in inter‐nest distance between individuals. Availability of nest sites due to snow melt patterns may be another factor influencing the distribution of nests at our site, as has been observed in other locations (Grabowski et al., 2013; Liebezeit et al., 2014), but the delay in nest initiation in 2019 relative to 2017 appears to be the primary response to delayed snow melt.

We cannot rule out the possibility that observed site differences in nesting distributions are related to varying detection probabilities across sites. Smith et al. (2009) conclude that variation in the probability of detection for shorebird nests is related primarily to species and stage of the nesting cycle, rather than site or density of nests. Conversely, detection ratios for nesting birds including shorebirds, based on the Arctic PRISM surveys, do suggest that nests at Canadian sites have slightly higher detection than in Alaska (Bart & Johnston, 2012). The methods for calculating detection, however, in both these cases are based on double sampling (and the assumption that intensive nest searching is effective), rather than other double survey methods (i.e., model‐based double survey), which have no underlying assumptions and are likely to yield more accurate estimates (McCaffery & Ruthrauff, 2004). Using the approach of hierarchical multi‐species occupancy modeling, detection probability of sandpiper nests at Karrak Lake (*p* = .01; 95% posterior interval: 0.06–0.15) is estimated to be much lower than anticipated based on previous studies (Dorazio & Royle, 2005; Kellett, 2021). If so, our estimates of nearest neighbor distance may actually be biased high, but further studies are needed to clarify the extent to which low detection rates influenced our findings. Studies evaluating the specific factors that affect detection at this site (and others) would be particularly helpful.

The observed aggregation of nests at Karrak Lake may also be influenced by natal site tenacity (i.e., distance from a bird's natal nest to the nest where it was first found breeding). Though there are few published measures of natal philopatry and natal site tenacity in sandpipers, and they are of limited geographic scope, available evidence suggests that natal site tenacity is correlated with the size of the nesting aggregation of origin; recruits from a small aggregation tend to nest closer to their hatch site than recruits from a large aggregation (Gratto et al., 1985; Jehl, 2006). Natal recruits settling in close proximity to the natal nest site could contribute to the apparent aggregation of nests, an idea that could be tested through ongoing marking and resighting or genetics studies.

### 4.2 Nest survival

Based on our findings, increasing local nest density did not confer a statistically significant advantage in daily nest survival, with only weak evidence (i.e., in one of 2 years) that such behavior may incur a cost. Similarly, we did not detect a significant effect of nesting in close proximity to a conspecific on nest survival, and together, our findings suggest that costs of aggregation may exist only under certain conditions. This is consistent with other studies, which present a generally mixed picture concerning the effects of local density on nest survival. Prairie‐breeding marbled godwits *Limosa fedoa*, for example, showed declining nest survival with increasing nest density, whereas willets *Tringa semipalmata inornata* did not, possibly due to variable impacts of predators, or availability of habitat, across study areas and years (Specht et al., 2020). Nest fate for Hudsonian godwits *Limosa haemastica* was not predicted by distance to nearest neighbor (Swift et al., 2017). For five smaller Arctic‐nesting shorebird species, there is some weak evidence that nest survival is lower with increasing relative abundance of active nests, although temporal variation in other factors, including parental behavior, appears to be more important (Smith & Wilson, 2010). For this same group of shorebirds, mean distance to nearest neighbor was not correlated with nest success (Smith et al., 2007). Saalfeld and Lanctot (2015) conclude that conservation efforts need to incorporate settlement pattern information related to nest densities throughout the range of Arctic breeding shorebirds.

We found evidence of a strong quadratic effect of nest age on survival in 2017, but not in 2019. This result is consistent with other studies that find variation in DSR as a function of temporal terms, including quadratic terms on either Season Day or Nest Age (Que et al., 2015; Smith & Wilson, 2010). Mid‐season declines in daily nest survival have been linked to variations in predator activity, dependent on the abundance of other nonshorebird prey (Mckinnon et al., 2014; Smith, 2009). At Karrak Lake, sandpiper nest survival and predator–prey interactions are likely further complicated by abundant nesting light geese, which can impact spatial variation in the occurrence of shorebird nest predators, occurrence of shorebird nests, and nest predation risks (Lamarre et al., 2017), and has also been shown to affect the selection of nesting habitat by other species breeding at Karrak Lake, including cackling geese and possibly king eider (Kellett & Alisauskas, 1997, 2011). Interannual variation in nest success is typically high in Arctic nesting shorebirds and 2 years of data are likely insufficient to make strong statements about the productivity of sandpipers at this site (Smith et al., 2007), yet our annual estimates of nest success are similar to results from other studies (Weiser et al., 2018; apparent annual survival = 0.65, *n* = 1210 nests), suggesting that sandpiper nests at Karrak Lake have relatively high survival, especially considering the active Arctic fox den on Camp Island in 2019. This population can provide important demographic data that may be in contrast to some sites in the eastern Canadian Arctic, where colonies have declined or disappeared entirely (Jehl, 2007). Our study thus adds to the growing body of evidence that an improved understanding of the demographic characteristics of central and eastern populations of this species, which are currently uncertain, is a key research need (Andres et al., 2012). Understanding drivers of nest success, as well as settlement patterns, in this species will make important contributions to conservation of the species.

## AUTHOR CONTRIBUTIONS

**Scott Freeman:** Conceptualization (lead); data curation (equal); formal analysis (lead); investigation (equal); methodology (equal); software (lead); validation (equal); visualization (equal); writing – original draft (lead); writing – review and editing (equal). **Katelyn Luff:** Investigation (equal); writing – review and editing (equal). **Kirsty Gurney:** Conceptualization (supporting); data curation (equal); formal analysis (equal); funding acquisition (lead); investigation (equal); methodology (equal); project administration (lead); resources (lead); supervision (lead); validation (equal); visualization (equal); writing – original draft (supporting); writing – review and editing (equal).

## FUNDING INFORMATION

Funding was provided by Environment and Climate Change Canada, with additional support for K. Luff through the Northern Scientific Training Program, Polar Knowledge Canada.

## CONFLICT OF INTEREST STATEMENT

There are no conflicts of interest in the publication of this manuscript for any author.

## PERMISSION TO REPRODUCE MATERIALS FROM OTHER SOURCES

None.

## Data Availability

Raw data and coding files used in this study are open access and available at https://data‐donnees.ec.gc.ca/data/species/scientificknowledge/aggregation‐of‐nests‐in‐an‐arctic‐breeding‐shorebird‐and‐daily‐survival‐rates/?lang=en.
